# Effectiveness of Patient Adherence Groups as a Model of Care for Stable Patients on Antiretroviral Therapy in Khayelitsha, Cape Town, South Africa

**DOI:** 10.1371/journal.pone.0056088

**Published:** 2013-02-13

**Authors:** Miguel Angel Luque-Fernandez, Gilles Van Cutsem, Eric Goemaere, Katherine Hilderbrand, Michael Schomaker, Nompumelelo Mantangana, Shaheed Mathee, Vuyiseka Dubula, Nathan Ford, Miguel A. Hernán, Andrew Boulle

**Affiliations:** 1 Centre for Infectious Disease Epidemiology and Research, School of Public Health and Family Medicine, University of Cape Town, Cape Town, South Africa; 2 Médecins sans Frontières, Cape Town, South Africa; 3 Khayelitsha Community Health Centre, Department of Health, Provincial Government of the Western Cape, Cape Town, South Africa; 4 Treatment Action Campaign, Cape Town, South Africa; 5 Médecins sans Frontières, Geneva, Switzerland; 6 Departments of Epidemiology and Biostatistics, Harvard School of Public Health, Boston, Massachusetts, United States of America; 7 Division of Health Sciences and Technology, Harvard-Massachusetts Institute of Technology, Boston, Massachusetts, United States of America; Johns Hopkins Bloomberg School of Public Health, United States of America

## Abstract

**Background:**

Innovative models of care are required to cope with the ever-increasing number of patients on antiretroviral therapy in the most affected countries. This study, in Khayelitsha, South Africa, evaluates the effectiveness of a group-based model of care run predominantly by non-clinical staff in retaining patients in care and maintaining adherence.

**Methods and Findings:**

Participation in “adherence clubs” was offered to adults who had been on ART for at least 18 months, had a current CD4 count >200 cells/ml and were virologically suppressed. Embedded in an ongoing cohort study, we compared loss to care and virologic rebound in patients receiving the intervention with patients attending routine nurse-led care from November 2007 to February 2011. We used inverse probability weighting to estimate the intention-to-treat effect of adherence club participation, adjusted for measured baseline and time-varying confounders. The principal outcome was the combination of death or loss to follow-up. The secondary outcome was virologic rebound in patients who were virologically suppressed at study entry. Of 2829 patients on ART for >18 months with a CD4 count above 200 cells/µl, 502 accepted club participation. At the end of the study, 97% of club patients remained in care compared with 85% of other patients. In adjusted analyses club participation reduced loss-to-care by 57% (hazard ratio [HR] 0.43, 95% CI = 0.21–0.91) and virologic rebound in patients who were initially suppressed by 67% (HR 0.33, 95% CI = 0.16–0.67).

**Discussion:**

Patient adherence groups were found to be an effective model for improving retention and documented virologic suppression for stable patients in long term ART care. Out-of-clinic group-based models facilitated by non-clinical staff are a promising approach to assist in the long-term management of people on ART in high burden low or middle-income settings.

## Introduction

Retaining patients in lifelong HIV care is a major challenge in many countries in sub-Saharan Africa, where antiretroviral treatment (ART) has been rapidly scaled up to some 5 million people as of the end of 2010. [1] In recent years in South Africa, an increasing proportion of patients on ART are being lost to follow-up (LTF) as overall the numbers on treatment increase. [2] Although up to a third of adult patients lost to care are estimated to have died, the majority are alive: without treatment, they are at increased risk of morbidity and mortality. [3].

Decentralization of services and task-shifting aspects of care to nurses and non-clinical staff, including patients, has been found to be feasible with good clinical outcomes.[4]–[12] However, such approaches are reaching their limits as increasing numbers of patients are initiated on ART. Accessible and flexible ART services that differentiate between the needs of clinically ill patients starting ART, and clinically stable patients who have been on ART for some time, have been suggested as important strategies for maintaining and improving retention and quality of care. [13].

Patient support groups have long been recognized as an important adjunct to clinical care that encouraged retention and adherence. [14] The key evolution in the re-emergence of patient groups is that they are now seen as an essential mechanism of service delivery, including dispensing of ART and symptom screening, and a means of decongesting formal health services, rather than being purely an adherence adjunct. While encouraging outcomes from these programs have been described, [7] they have yet to be formally evaluated against more clinically intensive models of care.

The number of people starting and retained on ART had progressively increased between 2001 and 2007 in a large urban ART clinic in Khayelitsha (Ubuntu Clinic), Cape Town, leading to overcrowding, longer waiting times during visits, and less time for counseling and clinical care of poorly adherent and newly enrolling patients, and for tracing of patients lost to follow-up. In response, in November 2007, patients stable on ART for at least 18 months or longer were offered voluntary participation in “adherence clubs” of 15–30 patients, which convened every two months facilitated by trained counselors, aiming to free up clinicians, decongest services and improve retention in and care and adherence.

This study evaluated the effectiveness of adherence clubs compared to traditional clinic-based care in maintaining or improving long-term retention-in-care and virologic suppression.

## Methods

### Type of Study

We developed a retrospective observational evaluation of adherence clubs. Accordingly, we built a Marginal Structural Model (MSM) using Inverse Probability of Treatment Weighting (IPTW) to estimate the intention-to-treat effect of adherence club participation. The analytical approach was developed specifically to address the obvious confounding bias whereby patients who were already doing well were more likely to be offered club participation. In the weighted analysis, club participation at any time following the start of the study was rendered independent of measured potential confounders. The study was nested within an ongoing cohort study of routine ART outcomes in Khayelitsha, Cape Town approved by the Human Research Ethics Committee of the University of Cape Town. [3] Individual patient consent was not needed, consistent with the South African Medical Research Council’s Guidelines on Ethics for Medical Research and the Declaration of Helsinki. Because this was a retrospective analysis of routine clinical service records, no additional data collection or procedures were undertaken from or on patients, all patient information was entered anonymously into the database using coded identification numbers, and no information that could reveal patient identity was entered into the database.

### Setting

ART was first offered in Khayelitsha in 2001 at three community health centers serving an estimated population of 400,000. In 2011, over 20,000 adults and children had started ART in the sub-district. The clinical protocols have been closely aligned with international and national guidelines since inception. At the time that the adherence clubs were first introduced, the first-line regimen comprised stavudine, lamivudine and nevirapine or efavirenz, and patients received twice-yearly viral load and CD4 count monitoring. In a national guideline revision in April 2010 tenofovir replaced stavudine in the starting regimen, and viral load and CD4 count monitoring was reduced to annually beyond the first year on ART. [15] Historically, the majority of consultations have been with nurses, while doctors have been available to see very ill patients or receive referrals. Given the large patient load of several thousand patients on ART, patients do not consistently see the same practitioner from visit to visit. Facility-based lay counselors have provided structured patient preparation and adherence training at ART initiation and when adherence challenges have been identified. Home visits and patient support groups in addition to routine clinical care were a feature of the program in the first three years, but were not available to the majority of patients subsequently.

### Adherence Clubs

Adherence clubs were piloted in late 2007 at the largest of the community health centers (Ubuntu Clinic) to reduce the burden on health services by shifting consultations and medicine collections for stable patients to “clubs”. These groups are facilitated by non-clinical staff (counselors), who have previously been trained in facility-based patient preparation and support, and who receive additional training and mentoring. The meeting times are scheduled outside of busy clinic times, and consequently are more convenient for patients with much shorter waiting times. The resulting decongestion of the clinic allows nurses to spend more time initiating new patients on ART or with ill patients or those experiencing difficulties.

Participation is offered to clinically stable adult patients who have been on ART for at least 18 months. Guidelines further recommend that patients should have had a CD4 count of more than 200 cells/µl in the previous six months and have had sustained viral load suppression. Groups of 15 to 30 patients are formed and convene at the clinics during quiet times such as the early mornings or afternoons. Medicines are pre-packaged for each participant and brought to the group by a counselor who weighs the patients and administers a symptom-based general health assessment. Any patients reporting symptoms suggestive of illness, adverse drug effects or who have weight loss are referred back to the clinic to be assessed by a nurse. The counselor or experienced patients lead short group discussions on a range of health and other topics requested by the club participants. A nurse attends these groups annually to draw blood for viral load and CD4 count testing.

As adherence clubs was a pilot, only some of the stable patients were offered participation, based on the clinician’s enthusiasm for the model, and the opening of new clubs: 20 clubs were established during the pilot. The determination of which stable patients were enrolled into the clubs was therefore largely driven by service factors as opposed to patient factors. Although not formally documented, most patients with the option to transition to club-based care took up this option due to the prospect of much less time spent waiting for medicines.

### Participants and Data Management

Data were extracted from the electronic medical record system used in the HIV clinics, which was updated daily by data capturers from structured clinical records completed by clinicians or from club registers completed by the club facilitators. Rule-based consistency assessments were used to identify specific patients and data elements for review by quality assurance staff.

Following the inclusion criteria that clinicians were using to offer club participation, we restricted the study to adult patients (≥18 years old) who had been on ART for at least 18 months when the pilot started, or who reached 18 months on ART during the study period, and who’s most recent CD4 count was above 200 cells/µl. Patients entered the analysis at their first eligible visit after November 1^st^, 2007 and exited at the date of outcome, date of censoring from follow-up or February 28^th^, 2011.

### Outcomes, Exposure and Confounders

The principal outcome was a combined outcome of time to either death or LTF. LTF was defined as not having any contact with the service in the six months following the analysis closure (between February 28^th^ and August 31^st^ 2011), and was determined to have happened at the date of the last contact with the service. Limited to patients who were virologically suppressed at study entry, the secondary outcome was the time to the first virologic rebound (>400 copies/mL).

**Figure 1:**
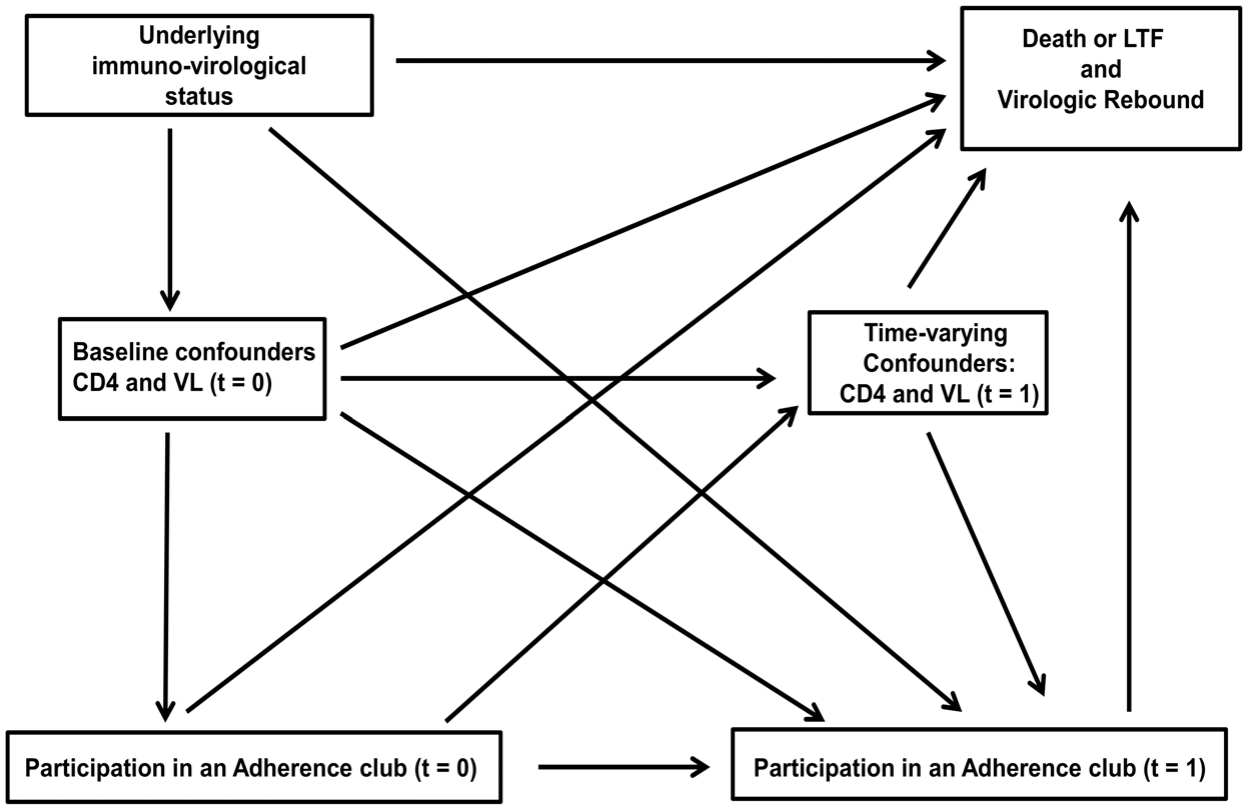
A directed acyclic graph representing the causal relationships between adherence club participation, outcomes, baseline and time-updated covariates.

The primary exposure/outcome relationships and variables considered *a priori* to be potential confounders are outlined in a causal diagram (Figure 1). These include age, gender, duration on ART and World Health Organization (WHO) clinical stage measured at study entry; CD4 count measured at ART initiation, study entry, at any given time (t), and t-6 months; and viral load suppression measured at study entry and at t.

### Statistical Analysis

We summarized patient characteristics as percentages for categorical variables and medians and interquartile ranges (IQR) for continuous variables. We computed crude rates for each outcome by patient characteristics at study entry, with 95% Poisson confidence intervals.

For each outcome, we used an weighted pooled logistic regression model to estimate the hazard ratio (HR) of club participation versus routine clinic-based care, including as covariates a time-varying indicator for history of club participation, month of follow-up (cubic splines with knots at the 5th, 25th, 50th, 75th and 95th percentiles) [16] and the baseline covariates (age in years, gender, CD4 at ART start and study entry per 100 cells/µl, Viral load suppression [<400 copies/ml] at study entry, duration on ART in months and WHO clinical stage). We used robust variance estimators.[17]–[19] The pooled logistic model closely approximates the Hazard Ratios because the probability of events occurring in any given month is small. [20], [21].

To estimate the inverse probability weights, we fitted a pooled logistic regression model for first participation in an adherence club. The model included month of follow-up, and the baseline and time-varying covariates (current CD4 count per 100 cells/µl, six month lagged CD4 count per 100 cells/µl and current viral load suppression <400 copies/ml). We used the predicted probabilities of club participation to compute the denominator of the IPTWs. The weights were then stabilized as previously described. [20] For the secondary outcome, the final stabilized weight included the probability of being uncensored by LTF.

Patients received a weight of one once they joined an adherence club, and were assumed to remain part of the club until the end of the study, akin to intention-to-treat analyses of randomized trials. The weighted analysis created a statistical pseudo-population in which the probability of entering an adherence club in each month was unrelated to the measured CD4 cell count or viral load, thus controlling for time-dependent confounders. [21] In order to show the magnitude of the measured time-varying confounding we compared the weighted hazard ratios for club participation to unweighted estimates for both outcomes, both adjusted for co-variates at study entry.

Finally, we explored the sensitivity of our estimates to alternative models specifications (categorizing continuous variables and including selected interaction terms) and truncation of the IPTWs. All analyses were performed with Stata v.12.

## Results

**Figure 2:**
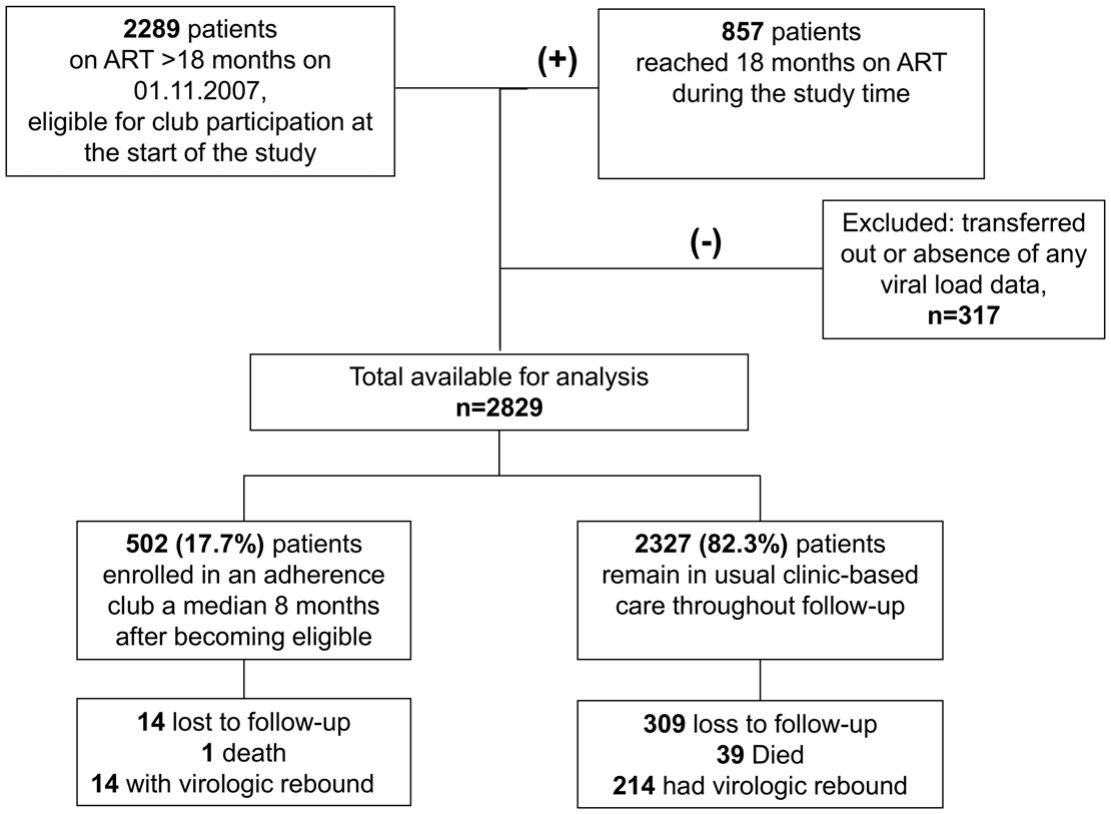
Patients included in the analysis, enrolment into clubs, and outcomes at the end of the study.

Of the 2829 individuals followed up for 8821 patient years (py), the median age of patients was 33 (IQR 29–39) years, and 71% were women (Table 1). Patients entered the study after a median 43 (IQR 28–61) months on ART, and with a median CD4 count of 202 cells/µl. The majority (88%) were virologically suppressed at study entry. Club participation was offered and accepted by 502 patients (Figure 2) who enrolled a median 8 (IQR 7–10) months after study entry, contributing 1273 py of follow-up. The frequency of available virologic monitoring was similar between club and other patients, averaging at least one test per year (Table S1).

**Table 1:** Patients characteristics at study entry and crude rates of progression to death/loss to follow-up and virologic rebound by club participation.

			Event rates (per 1000 person-years, 95% CI)					
			Death/loss to follow-up			Virologic rebound*		
		N (%)	In club**	Not in club	N (%)*	In club**	Not in club	
All patients combined		2829	29.8 (17.9–49.5)	116.9 (105.2–129.8)	2517	31.8 (18.8–53.6)	90.4 (79.1–103.4)	
Age in years at study entry								
<25		284 (10.0)	30.8 (4.3–218.6)	192.8 (149.4–248.9)	228 (9.1)	68.0 (17.0–271.9)	151.4 (106.5–215.3)	
25–34		1397(49.4)	18.8 (7.8–45.3)	120.6 (104.0–139.9)	1234 (49.0)	34.9 (17.5–69.9)	97.5 (80.9–117.4)	
35–44		841 (29.7)	30.1 (12.9–74.4)	82.1 (65.3–103.3)	778 (30.9)	20.8 (6.7–64.6)	72.4 (55.4–94.5)	
≥45		307 (10.9)	91.9 (34.5–244.9)	123.1 (90.6–167.2)	277 (11.0)	26.3 (3.7–186.5)	65.1 (41.0–103.4)	
Median (IQR)		32.9 (28.5–39.0)			33.2 (28.7–39.3)			
Gender								
Male		831 (29.4)	41.5 (18.6–92.3)	123.1 (101.9–148.8)	749 (29.8)	7.8 (1.1–55.8)	75.2 (57.5–98.4)	
Female		1997 (70.6)	25.1 (13.1–48.3)	114.3 (100.7–129.7)	1767 (70.2)	41.5 (24.1–71.5)	96.9 (83.1–113.2)	
CD4 count (cells/µl) at ART start								
<50		601 (21.2)	15.3 (3.8–61.0)	153.3 (124.6–188.4)	518 (20.6)	34.2 (12.8–91.3)	94.6 (69.9–128.1)	
50–99		560 (19.8)	34.2 (12.8–91.2)	134.1 (107.3–167.7)	496 (19.7)	30.0 (9.6–93.0)	84.6 (61.6–116.3)	
100–199		1276 (45.1)	37.8 (18.9–75.5)	100.8 (85.3–119.1)	1151 (45.4)	27.1 (11.3–65.1)	96.2 (79.5–116.4)	
≥200		392 (13.4)	23.3 (3.3–165.7)	96.0 (71.1–129.2)	352 (14.0)	51.1 (12.8–204.5)	75.4 (52.0–109.2)	
Median (IQR)		121 (60–176)			124 (62–177)			
CD4 count (cells/µl) at study entry								
<50		520 (18.4)	–	254.2 (216.5–298.4)	426 (16.9)	–	106.7 (79.7–142.9)	
50–99		203 (7.2)	–	101.3 (68.9–148.7)	166 (6.6)	–	64.5 (37.4–110.9)	
100–199		675 (23.9)	52.4 (7.4–372.0)	71.3 (55.3–91.7)	582 (23.1)	235.1 (75.8–728.8)	78.3 (59.8–102.5)	
≥200		1431 (50.6)	29.6 (17.5–49.9)	87.3 (72.6–105.0)	1343 (53.4)	26.3 (14.5–47.5)	96.6 (79.7–117.2)	
Median (IQR)		202 (97–386)			215 (110–404)			
Duration on ART in months at study entry								
<24		510 (18.1)	–	136.3 (109.9–168.9)	464 (18.4)	–	47.9 (32.6–70.3)	
25–48		1084 (38.3)	76.7 (19.2–306.9)	132.9 (114.3–154.5)	961 (38.2)	–	90.6 (73.9–111.1)	
>48		1235 (43.6)	27.3 (15.9–47.1)	87.2 (71.2–106.5)	1092 (43.4)	33.7 (19.9–56.9)	119.2 (97.5–145.7)	
Median (IQR)		43.1 (28.0–61.1)			42.8 (27.7–60.9)			
Virologic suppression at study entry								
Yes		2501 (88.4)	28.6 (16.9–48.3)	110.6 (98.6–124.2)	–	–	–	
No		327 (11.6)	74.9 (10.5–531.5)	160.0 (124.1–205.9)	–	–	–	
WHO clinical stage at study entry								
I/II		781 (27.6)	32.2 (10.4–100.3)	81.6 (64.7–102.8)	716 (28.5)	36.9 (11.9–114.4)	79.5 (61.6–102.7)	
III/IV		2045 (72.4)	29.3 (16.6–51.5)	131.9 (117.2–148.4)	1789 (71.5)	30.6 (16.9–55.3)	95.6 (81.7–112.0)	

* Restricted to patients who had virologic suppression at study entry, n = 2517.

** Patients who went on to enrol in a club contributed analysis time to the “not in club” group until they were enrolled in the club. There is no single point in time where characteristics of patients who enrol in clubs and other patients can be formally compared due to the progressive nature of club enrolment. Instead predictors of club participation are presented in Table 2. Table 1 shows event rates since study entry to the outcome endpoint or censoring.

ART: Antiretroviral therapy; WHO: World Health Organization; CI: Confidence interval; IQR (inter-quartile range).

By the end of the study, 12.8% of patients were LTF or had died (323 LTF and 40 deaths), and 9.0% had virologic rebound. Both outcomes were less frequent for patients participating in the clubs (29.8 vs 116.8 per 1000 py for LTF/death, crude rate ratio [RR] 0.25, 95% CI 0.14–0.41 and 31.8 vs 90.4 per 1000 py for virologic rebound, RR 0.35, 95% CI 0.31–0.40, Table 1).

Overall, patients with lower CD4 counts at study entry, viremia and clinical stage III/IV had higher crude rates of death or LTF, but this risk was higher in patients not enrolled in clubs. Patients in normal clinic-based care who were <25 years old and who entered the study with CD4 counts <50 cells/µl had the highest rates of death or LTF (192.8 and 254 per 1000 py respectively, Table 1).

Virologic rebound was similarly high in patients <25 years not participating in a club (151.4 per 1000 py). Longer durations on ART were associated with higher rates of virologic rebound, but lower rates of death or LTF (Table 1).

Club participation was strongly associated with virologic suppression (<400 copies/ml) at study entry (HR 3.1, 95% CI 1.3–7.6), and during subsequent follow-up (HR 4.5, 1.8–12.5, Table 2). Patients with higher CD4 counts at study entry, an increasing CD4 count during follow-up, women, and patients who had been on ART for longer were also more likely to be enrolled in a club. For the secondary analysis restricted to virologically suppressed patients, the remaining associations with club participation were comparable with the primary analysis (Table 2). The estimated IPTWs had means of 1.06 and 1.05 respectively (Table S2, Figure S1).

**Table 2:** Factors associated with adherence club enrolment.

	Death or LTF		Virologic rebound*	
Covariates at study entry	HR (95% CI)**	P-value†	HR (95% CI)**	P-value†
Age in years	1.00 (0.98–1.02)	0.733	1.00 (0.98–1.01)	0.718
Gender (males vs females)	0.75 (0.60–0.93)	0.010	0.72 (0.58–0.91)	0.006
CD4 count at ART start (per 100 cells/µl)	0.86 (0.76–0.96)	0.012	0.83 (0.73–0.94)	0.005
CD4 count at study entry (per 100 cells/µl)	1.85 (1.70–2.00)	<0.001	1.80 (1.65–1.95)	<0.001
Viral load suppression at study entry (<400 copies/ml)	3.06 (1.25–7.60)	0.016		
Duration on ART (per 12 months)	1.52 (1.43–1.61)	<0.001	1.53 (1.43–1.62)	<0.001
WHO clinical stage (I/II vs III/IV)	0.85 (0.66–1.11)	0.214	0.86 (0.66–1.12)	0.281
Time-varying covariates				
Current CD4 count (Per 100 cells/µl)	1.02 (0.94–1.09)	0.585	1.01 (0.93–1.09)	0.754
Six month lagged CD4 count (Per 100 cells/ml)	0.62 (0.57–0.68)	<0.001	0.64 (0.58–0.70)	<0.001
Current viral load <400 copies/ml	4.46 (1.79–12.5)	0.001		

* Restricted to patients who had virologic suppression at study entry, n = 2517.

** Hazard ratios were derived from a pooled unweighted logistic regression model fit on the subsample of person-months of follow-up for which no club participation had yet occurred through the previous months.

† P-value based on Wald test.

LTF: Loss to follow-up; ART: Antiretroviral therapy; WHO: World Health Organization; HR: Hazard Ratio; CI: Confidence interval.

**Figure 3:**
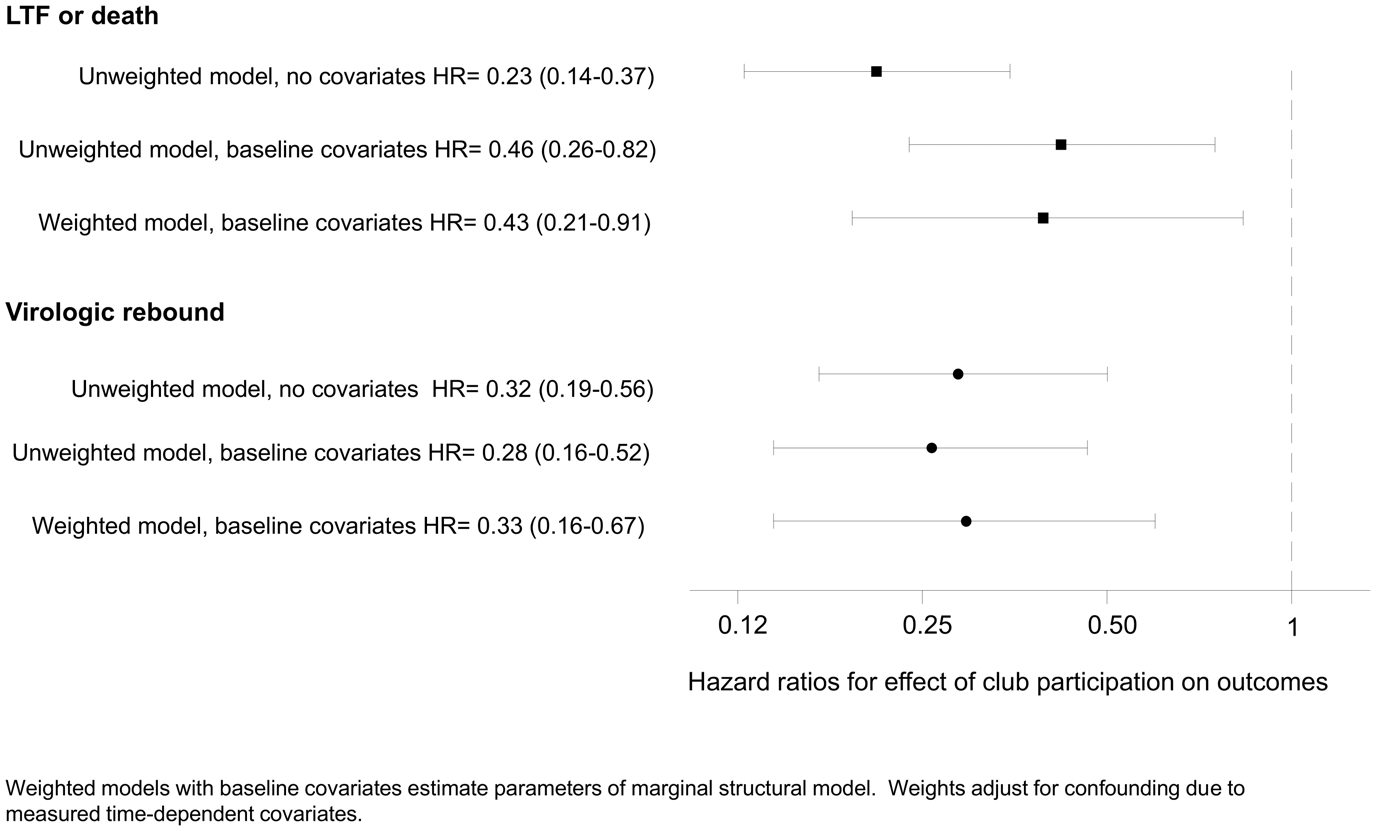
Estimated effect of club participation from unweighted and weighted Cox models. Weighted model with baseline covariates estimates the parameters of a marginal structural model, adjusting for confounding due to measured time-dependent covariates.

In the final weighted analysis (MSM) club participation reduced death or loss to follow-up (HR = 0.43, 95% CI 0.21–0.91) and virologic rebound (HR = 0.33, 95% CI 0.16–0.67). For both outcomes, there was little evidence of time-varying confounding by CD4 count and viral load, as weighted and unweighted models resulted in similar estimates (Figure 3).These estimates were stable in sensitivity analyses (Table S2).

Although other associations with each outcome were not causally considered, when adjusting the final models for other covariates at study entry, younger patients appeared at greater risk of both outcomes as did patients with lower CD4 counts (Table S3).

## Discussion

During the study period the delivery of ART and adherence support for stable adult patients on long-term ART in Khayelitsha was shifted from individual consultations with nurses to group consultations led by non-clinical staff. This model of care resulted in improved retention on ART and decreased rates of virologic rebound.

The imperative for simplified models of care for ART in high burden countries is well established, [13] and is especially relevant in the context of treatment as prevention [22], [23] which would require even greater expansion of ART provision. There is now a solid body of evidence demonstrating that task-shifting has been successfully implemented in a variety of settings and models, expanding access to care, improving or maintaining quality of care or improving cost-effectiveness. [24] The majority of the studied models have involved the shifting of key care tasks to less qualified clinical staff or to non-clinical staff, but few have been based on patient groups.

Currently there is an emerging interest in patient groups as the vehicle of service delivery, and not just as an adherence adjunct to care. [7] Administrative efficiency and decongestion of services are key aspects of the model: patients can bypass queues for collecting folders, waiting to see a practitioner, and waiting for medicines, converting a consultation process that previously could take an entire day into one that can be completed within half an hour.

### Retention in Care

Improved retention in care might result due to the removal of these and other structural barriers to care, [25], [26] which are inherently improved with out-of-clinic models. Patients may also potentially take responsibility for tracing and linking group members back to care when they miss visits, and arguably be more successful at achieving this than facility staff. Finally the group dynamic itself may be an important contributor as was historically motivated. [14].

A previous descriptive evaluation of a patient-group-based model of care in Mozambique reported 97.5% of patients retained in self-forming patient groups, [7] which is similar to the proportion retained in the adherence clubs in Khayelitsha.

### Virologic Rebound

Virologic rebound was expectedly low in both groups due to the restriction to stable patients, but was lower in the club model. There is a strong association between virologic rebound and gaps or delays in medication collection. [27] The ease of medicine collection associated with club participation (patients collect pre-packaged medications during the club) and strong incentives to collect medicines at the time of scheduled club meetings could reduce potential gaps in treatment collection. In routine facility-based care the time spent at the facility sometimes precludes patients from keeping appointments due to other commitments.

Although the main motivation for the adherence club model was the need for more efficient care, the potential for the clubs to function as peer support groups is an important consideration. The value of support groups is widely argued, but the impact on virologic outcomes has not been systematically assessed. [28].

### Additional Findings

We have previously reported higher rates of loss to follow-up and virologic failure in adolescents and young adults in the study setting, [3], [29] consistent with the findings when adjusting our analyses by age, notwithstanding that the study was not explicitly designed to assess these associations. These findings and the specific challenges in supporting adolescents and young adults have also been noted elsewhere. [30].

### Strengths and Limitations of the Study

The use of a combined outcome for LTF and death was necessitated due to the high proportion of unrecorded deaths that end up being defined as LTF, [3] but precluded the exploration of each process separately. Although the completeness of viral load results was similar between groups, the completeness was less than would be anticipated based on guidelines, possibly reducing the overall ascertainment of virologic rebound.

The majority of patients transitioning to club-based care were already responding well to therapy and were assumed to be adherent based on immunologic and virologic response. This indication bias when comparing to patients remaining in routine care was addressed in the analysis in two ways. First the analysis was adjusted for the same variables considered by clinicians when inviting patients to join a club, measured at the time that patients first became eligible for the clubs. Secondly, through the inverse probability weighting, any time varying confounding that might result from changes in these parameters after study entry was further adjusted for. Although simpler cohort analytical approaches showed similar benefits (as evidenced by the presented unweighted models), the approach based on IPTW’s was preferred due to the strong prior assumption that time-dependent confounding may be present. Assuming relevant associations with club participation have been correctly measured and included in the analysis, and there is no unmeasured confounding, the weighted results should approximate those from a randomized comparison. [20].

As with all observational studies, we cannot rule out insurmountable limitations, especially in adjusting for biases in who accessed adherence clubs. There remains the possibility that unmeasured patient-level factors such as openness to group-based care, and willingness and motivation to participate in the clubs could result in residual confounding. As described however, the determination of who entered clubs and who did not was largely the result of clinician practice and club availability rather than patient preference overwhelmingly patients offered the club model took up the option due to the perceived benefits. It remains a limitation of the study however that refusals and which club entries were patient-initiated were not recorded, and reasons for refusal where present could have introduced unmeasured confounding.

This analysis equates to an intention-to-treat study as after first entering a club, patients were considered to be in the club treatment group until the end of the study. This is necessitated as a return to clinic-based routine care is usually the result of clinical or adherence problems, which would increase the event rates in routine care if this follow-up time was apportioned to routine care, and bias the analysis in favour of the club model.

Finally, we were unable to formally assess potential risks associated with non-clinical care. While it is reassuring that retention and adherence were likely improved by the club participation, it is possible that some clinical diagnoses were missed.

### Future Research

Club availability has since been expanded and extended to all clinics in the sub-district, enabling ongoing increases in enrolment without additional clinical staff. Future operational research will need to verify that adherence strategies based on patient groups can be successfully adapted to the specific needs and characteristics of different service and community settings. The management of these groups is becoming increasingly challenging as the number of groups associated with a single clinic increases, requiring new management strategies and related research. Groups are now meeting in the community, introducing challenges in the delivery of pre-packaged drugs to community settings, and the collection, transfer and review of patient and program management data.

### Conclusion

Patient adherence groups were found to be an effective model for improving retention and documented virologic suppression for stable adult patients in long term ART care in Khayelistha. Models based on patient adherence groups meeting outside of pressurized clinical consultation areas, and facilitated by non-clinical staff, are a promising approach to assist in the next wave of increased access to HIV treatment.

## Supporting Information

Figure S1 **Summary of the stabilised inverse probability weight distribution by study duration.**
(TIF)

Table S1 **Virologic endpoints and availability of viral load measurements to the analysis by club participation and calendar year.**
(DOC)

Table S2 **Effect of club participation on the risk of death or loss to follow-up and virologic rebound under progressive truncation of inverse probability weights and alternative model specifications, n = 2829.**
(DOC)

Table S3 Associations* between co-variates at study entry and death/loss to follow-up and virologic rebound.
(DOC)
