# Supplementary material for: Effectiveness of Patient Adherence Groups as a Model of Care for Stable Patients on Antiretroviral Therapy in Khayelitsha, Cape Town, South Africa
Source: PLoS One. 2013 Feb 13;8(2):e56088. doi: 10.1371/journal.pone.0056088 (PMC3571960; doi:10.1371/journal.pone.0056088)
Supplement: Table S1 — Virologic endpoints and availability of viral load measurements to the analysis by club participation and calendar year. (DOC) [file pone.0056088.s002.doc]

**Table S1**. Virologic endpoints and availability of viral load measurements to the analysis by club participation and calendar year

|  | Adherence clubs (n= 497), n(%) |  | Normal clinic-based care (n= 2020), n(%) |  | Total number of patients (n = 2517), n(%) |
| --- | --- | --- | --- | --- | --- |
| Virologic endpoint* |  |  |
|  |  |
|  |  |  |  |  |  |
| Virological rebound | 14 (2.8) |  | 214 (10.6) |  | 228 (9.1) |
|  |  |  |  |  |  |
|  | Average number of virologic tests performed by patient and year | | | | |
| Year | Adherence clubs (n= 502) |  | Normal clinic-based care (n= 2327) |  | The whole cohort (n= 2829) |
|  |  |  |
| 2008 | 1.2 |  | 1.0 |  | 1.1 |
| 2009 | 1.3 |  | 1.2 |  | 1.2 |
| 2010 | 1.0 |  | 0.9 |  | 1.0 |
| *Restricted sample including those patients who had virologic suppression at study entry , n= 2517 | | | | | |
