# Supplementary material for: Effectiveness of Patient Adherence Groups as a Model of Care for Stable Patients on Antiretroviral Therapy in Khayelitsha, Cape Town, South Africa
Source: PLoS One. 2013 Feb 13;8(2):e56088. doi: 10.1371/journal.pone.0056088 (PMC3571960; doi:10.1371/journal.pone.0056088)
Supplement: Table S2 — Effect of club participation on the risk of death or loss to follow-up and virologic rebound under progressive truncation of inverse probability weights and alternative model specifications, n = 2829. (DOC) [file pone.0056088.s003.doc]

**Table S2**. Effect of club participation on the risk of death or loss to follow-up and virologic rebound under progressive truncation of inverse probability weights and alternative model specifications, n= 2829

|  |  | Estimated weights for the outcome Death or LTF | | |  | Estimates of effect of club on Death or LTF | |  | Estimated weights for the outcome virologic rebound* | | |  | Estimates of effect of club on virologic rebound* | |
| --- | --- | --- | --- | --- | --- | --- | --- | --- | --- | --- | --- | --- | --- | --- |
|  |  |  |  |  |
| Truncation percentiles |  | Mean (SD) |  | Minimum/  maximum |  | Estimate | Standard Error |  | Mean (SD) |  | Minimum/maximum |  | Estimate | Standard Error |
|  |  |  |  |  |  |
|  |  |  |  |  |  |  |  |  |  |  |  |  |  |  |
| 0,100 |  | 1.06 (0.54) |  | 0.01/11.12 |  | 0.43 | 0.16 |  | 1.05 (0.44) |  | 0.01/10.4 |  | 0.32 | 0.12 |
| 1,99 |  | 1.09 (0.42) |  | 0.05/3.02 |  | 0.44 | 0.14 |  | 1.07 (0.34) |  | 0.56/2.60 |  | 0.33 | 0.11 |
| 5,95 |  | 1.01 (0.07) |  | 0.92/1.22 |  | 0.44 | 0.13 |  | 1.02 (0.07) |  | 0.91/1.20 |  | 0.30 | 0.09 |
| 10,90 |  | 1.01 (0.03) |  | 0.97/1.07 |  | 0.45 | 0.13 |  | 1.00 (0.03) |  | 0.96/1.06 |  | 0.30 | 0.09 |
| 25,75 |  | 1.00 (0.01) |  | 0.99/1.01 |  | 0.46 | 0.13 |  | 1.00 (0.01) |  | 0.99/1.01 |  | 0.29 | 0.08 |
|  |  |  |  |  |  |  |  |  |  |  |  |  |  |  |
| * Restricted sample including those patients who had virologic suppression at study entry , n= 2517  LTF: Loss to follow-up; ART: Antiretroviral therapy; WHO: World Health Organization; HR: Hazard Ratio; CI: Confidence interval; VL: Viral load  **Alternative model specifications**  **Principal outcome: Death or loss to follow-up**  Categoric model (Age, CD4 at ART and study entry): HR 0.41(0.22-0.76) SE (0.130)  Interaction of current VL and time (month): HR 0.43 (0.20-0.90) SE (0.163)  **Secondary outcome: Virologic rebound**   Categoric model (Age, CD4 at ART and study entry): HR 0.32 (0.17-0.58) SE (0.098)   Interaction current CD4 and time (month): HR 0.31 (0.16-0.62) SE (0.108) | | | | | | | | | | | | | | |
