# Supplementary material for: Effectiveness of Patient Adherence Groups as a Model of Care for Stable Patients on Antiretroviral Therapy in Khayelitsha, Cape Town, South Africa
Source: PLoS One. 2013 Feb 13;8(2):e56088. doi: 10.1371/journal.pone.0056088 (PMC3571960; doi:10.1371/journal.pone.0056088)
Supplement: Table S3 — Associations* between co-variates at study entry and death/loss to follow-up and virologic rebound. (DOC) [file pone.0056088.s004.doc]

**Table S3.** Associations* between co-variates at study entry and death/loss to follow-up and virologic rebound.

|  |  | Death or loss to follow-up (N=2829) | | |  | Virologic rebound* (N=2517) | | |
| --- | --- | --- | --- | --- | --- | --- | --- | --- |
|  |  | Death or LTF, n(%) | Person-years at risk (per 1000) | Unweighted adjusted** HR (95%CI) |  | Virologic rebound*, n(%) | Person-years at risk (per 1000) | Unweighted adjusted** HR (95%CI) |
|  |  |  |
|  |  |  |  |  |  |  |  |  |
| Age in years at study entry |  |  |  |  |  |  |  |  |
|  | <25 | 60 (21.2) | 338 | 1.73 (1.15-2.61) |  | 33 (14.5) | 234 | 2.29 (1.28-4.10) |
|  | 25-34 | 180 (12.8) | 1716 | 1.00 (0.71-1.39) |  | 119 (9.6) | 1367 | 1.49 (0.92-2.41) |
|  | 35-44 | 78 (9.4) | 1050 | 0.65 (0.44-0.94) |  | 57 (7.3) | 890 | 1.06 (0.63-1.78) |
|  | ≥45 | 45 (14.6) | 376 | 1 |  | 19 (6.9) | 314 | 1 |
| Gender |  |  |  |  |  |  |  |  |
|  | Female | 250 (12.5) | 2466 | 0.95 (0.78-1.15) |  | 174 (9.8) | 1973 | 0.99 (0.97-1.02) |
|  | Male | 113 (13.5) | 1013 | 1 |  | 54 (7.2) | 831 | 1 |
| CD4 count (cells/μl) at ART start |  |  |  |  |  |  |  |  |
|  | ≥200 | 44 (11.2) | 491 | 0.94 (0.61-1.47) |  | 30 (8.5) | 410 | 1.12 (0.67-1.87) |
|  | 100-199 | 146 (11.4) | 1580 | 1.53 (1.04-2.24) |  | 111 (9.6) | 1286 | 1.06 (0.71-1.58) |
|  | 50-99 | 81 (14.5) | 690 | 1.74 (1.17-2.60) |  | 41 (8.3) | 549 | 1.08 (0.65-1.79) |
|  | <50 | 92 (15.3) | 718 | 1 |  | 46 (8.9) | 560 | 1 |
| CD4 count (cells/μl) at study entry |  |  |  |  |  |  |  |  |
|  | ≥200 | 127 (8.9) | 1767 | 0.38 (0.28-0.51) |  | 114 (8.5) | 1483 | 0.79 (0.53-1.17) |
|  | 100-199 | 61 (8.9) | 861 | 0.19 (0.12-0.29) |  | 56 (9.6) | 689 | 1.00 (0.61-1.79) |
|  | 50-99 | 26 (12.8) | 257 | 0.22 (0.13-0.38) |  | 13 (7.8) | 202 | 0.79 (0.38-1.62) |
|  | <50 | 149 (28.6) | 595 | 1 |  | 45 (10.6) | 431 | 1 |
| Virologic suppression (<400 copies/ml) at study entry |  |  |  |  |  |  |  |  |
|  | Yes | 302 (12.0) | 3091 | 0.77 (0.57-1.00) |  | - | - | - |
|  | No | 61 (18.7) | 388 | 1 |  | - | - | - |
| Duration on ART in months at study entry |  |  |  |  |  |  |  |  |
|  | ≤24 | 83 (16.1) | 610 | 1 |  | 26 (5.6) | 544 | 1 |
|  | 25-48 | 172 (15.9) | 1305 | 0.68 (0.50-0.92) |  | 93 (9.7) | 1050 | 1.85 (1.20-2.84) |
|  | >48 | 108 (8.7) | 1565 | 0.32 (0.21-0.48) |  | 109 (10.0) | 1212 | 2.56 (1.52-4.33) |
| WHO clinical stage at study entry |  |  |  |  |  |  |  |  |
|  | I/II | 75 (9.4) | 975 | 0.53 (0.40-0.70) |  | 62 (8.7) | 823 | 0.89 (0.64-1.24) |
|  | III/IV | 288 (14.1) | 2502 | 1 |  | 166 (9.2) | 1979 | 1 |
|  |  |  |  |  |  |  |  |  |
| ART: Antiretroviral therapy; CI: Confidence interval; HR: Hazard Ratio; WHO: World Health Organization | | | | |  |  |  |  |
| * The study was not designed to explicitly explore causal associations between the listed covariates in this table and the outcomes.  Theses associations are therefore presented separately to the main casually considered associations between club participation and each outcome,  although they are additionally adjusted for club participation. | | | | | | | | |
| * Restricted to patients with virologic suppression at study entry | | | | |  |  |  |  |
| **Adjusted by club participation and covariates at baseline | | | | | |  |  |  |
